# Monitoring of central venous pressure and stroke volume variation in a case with a ruptured brain arteriovenous malformation and Fontan circulation

**DOI:** 10.1186/s40981-017-0089-2

**Published:** 2017-04-26

**Authors:** Toshiyuki Nakanishi, Kazuyoshi Ishida, Kiyotaka Shiramoto, Mishiya Matsumoto

**Affiliations:** 1 Department of Anesthesiology, Yamaguchi University Graduate School of Medicine, 1-1-1 Minami-Kogushi, Ube, Yamaguchi 755-8505 Japan; 2 Present Address: Department of Anesthesiology, Japan Community Healthcare Organization Tokuyama Central Hospital, 1-1, Koda-cho, Shunan, Yamaguchi 745-8522 Japan

**Keywords:** Fontan circulation, Hypoplastic left heart syndrome, Central venous pressure, Stroke volume variation, Fluid resuscitation

## Abstract

**Background:**

Patients with complex congenital heart disease increasingly undergo noncardiac surgeries because of advancements in surgical techniques and medical management. In Fontan circulation, maintaining an adequate transpulmonary gradient is essential for preserving both pulmonary blood flow and cardiac output. However, intraoperative circulatory monitoring of Fontan patients has not been established.

**Case presentation:**

A 17-year-old girl required an emergency craniotomy for ruptured arteriovenous malformation. She had a surgical history of bidirectional Glenn operation and Fontan palliation for her congenital hypoplastic left heart syndrome and double outlet right ventricle. We performed general anesthesia with continuous monitoring of central venous pressure (CVP) and stroke volume variation (SVV). Transesophageal echocardiography was not conducted because of difficulty in manipulating the probe due to the patient’s position and surgical setting. After incision of the dura, approximately 1700 ml of rapid blood loss from the arteriovenous malformation was observed in 30 min. In this period, CVP decreased from 15 to 5 mmHg or less. In contrast, there was only a slight rise in SVV from 5 to 8%. We rapidly administered fluid and then transfused blood, and CVP gradually recovered to 10–15 mmHg. During the surgery, circulatory collapse was not observed. The patient was transferred to the intensive care unit under sedation and controlled ventilation.

**Conclusions:**

CVP decreased sharply, whereas SVV rose only slightly during acute bleeding in the present case. CVP monitoring may have been useful for the management of an acute bleeding case with a Fontan circulation but SVV may not have been reliable. As more patients with a Fontan circulation undergo noncardiac surgeries, appropriate circulatory monitoring in these patients should be further investigated.

## Background

Advancements in surgical techniques and medical management have improved the survival rate of patients with congenital heart disease, who increasingly undergo noncardiac surgeries [1]. The Fontan operation, a palliative procedure for single ventricle defects, places the systemic and pulmonary circulations in series, driven by a single ventricular chamber [2]. In the Fontan circulation, maintaining an adequate transpulmonary gradient is essential for preserving both pulmonary blood flow and cardiac output [3]. However, an adequate preload and low pulmonary vascular resistance that are required to maintain the transpulmonary gradient can be jeopardized under general anesthesia because of anesthetics and positive pressure ventilation.

Some investigators have reported successful perioperative management of Fontan cases using central venous pressure (CVP), which represents mean pulmonary arterial pressure in these patients [4–6]. Recently, less invasive hemodynamic monitoring such as arterial pressure-based cardiac output and stroke volume variation (SVV) has become available for managing patients with hemodynamic instability [7]. However, it has not been determined whether less invasive monitoring aids the management of patients with a Fontan circulation. Here, we report the anesthetic management of a patient with a Fontan circulation who underwent emergency craniotomy for ruptured arteriovenous malformation (AVM) by continuously measuring cardiac index (CI) and SVV calculated with a FloTrac Sensor (Edwards Lifesciences, Irvine, CA) as well as CVP.

## Case presentation

A 17-year-old girl with a height of 150 cm and weight of 55 kg was referred to our hospital for disturbance of consciousness and right hemiparesis. She was diagnosed with acute subdural and subcortical hemorrhage due to ruptured AVM, and emergency craniotomy was performed under general anesthesia. She had a surgical history of left Blalock–Taussig shunt at the age of 2 months, bidirectional Glenn operation at the age of 3 years, and Fontan palliation (extracardiac conduit total cavopulmonary connection without fenestration of the atrium) at the age of 5 years for her congenital hypoplastic left heart syndrome (HLHS), double outlet right ventricle, atrioventricular septal defect, pulmonary atresia, and absence of inferior vena cava. She had received aspirin, warfarin, propranolol, imidapril, furosemide, and spironolactone preoperatively. Her activities of daily living were maintained. Preoperative transthoracic echocardiography (TTE) showed normal systolic and diastolic function of the univentricular chamber with trivial atrioventricular valve regurgitation.

In addition to the American Society of Anesthesiologists’ standard monitors, monitoring of arterial blood pressure (ABP), CI, and SVV was started via the left radial artery after confirming the equivalence of non-invasive blood pressure on her right arm. After induction of general anesthesia using target-controlled infusion of propofol at 3 μg/ml, remifentanil infusion at a rate of 0.2 μg/kg/min, and 40 mg of rocuronium, tracheal intubation was performed. Positive pressure ventilation was started with respiratory rate at 16 breaths/min, tidal volume 330 ml, I:E ratio 1:2, positive end-expiratory pressure 0 cmH_2_O, and peak airway pressure 20 cmH_2_O. We inserted a central venous catheter via the right internal jugular vein under X-ray fluoroscopy to a length of 11 cm and started continuous CVP monitoring. After positioning the CVP transducer at the same height as the patient’s heart, the initial value of CVP was 15 mmHg; we therefore attempted to maintain this value intraoperatively.

**Fig. 1:**
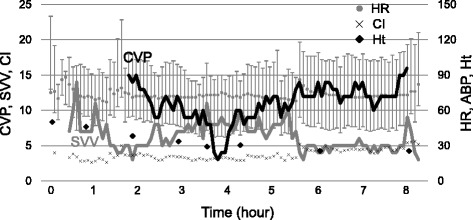
Intraoperative changes of hemodynamic parameters. Changes in CVP (*black line*) and SVV (*gray line*). High and low lines show systolic and diastolic blood pressure. CVP = central venous pressure, SVV = stroke volume variation, CI = cardiac index, HR = heart rate, ABP = arterial blood pressure, Ht = hematocrit

After the incision of the dura, rapid bleeding from the AVM was observed, and CVP suddenly decreased to less than 5 mmHg (Fig. 1). We first rapidly administered 500 ml of crystalloid, 500 ml of colloid, and 720 ml of fresh frozen plasma. We did not transfuse packed red blood cells at this time because high values of hematocrit (approximately 45%) were observed preoperatively. After that, approximately 1000 ml of blood loss was observed and hematocrit decreased to 29%. Then, we started to administer packed red blood cells to prevent an excess decrease in hematocrit. After fluid and blood transfusion, CVP gradually recovered to 10–15 mmHg. Approximately 1700 ml of rapid blood loss was observed in 30 min. Low CVP values (<5 mmHg) continued for approximately 20 min. In contrast, there was only a slight rise in SVV from 5 to 8%. CI was kept within an acceptable range (2.9–3.5 l/min/m^2^). Heart rate (HR) and ABP were also maintained without the use of inotropes or vasopressors. AVM resection and external decompression ended with a total operation time of 383 min, and the patient was transferred to the intensive care unit under sedation and controlled ventilation. In total, 1960 ml of blood loss and 540 ml of urine volume were observed. A total of 2500 ml of crystalloid and 800 ml of colloid were administered, and 840 ml of packed red blood cells, 1680 ml of fresh frozen plasma, and 400 ml of platelets were transfused.

Three days after surgery, the patient was still under controlled ventilation, but her hemodynamic state and circulatory monitoring values were stable (HR 75 bpm, ABP 110/50 mmHg, CVP 13 mmHg, CI 4.5 l/min/m^2^, and SVV 5%). At that time, ventricular systolic function and diastolic dimension measured by TTE were similar to those observed before surgery.

## Discussion

An acute change in CVP but not SVV due to acute blood loss secondary to AVM rupture was observed in this case with a Fontan circulation. To our knowledge, no report has described a sharp decrease in CVP due to acute bleeding and subsequent fluid resuscitation in Fontan cases.

Perioperative high values of CVP were observed in previous reports of Fontan cases (13–23 mmHg) [4–6]. We believe that patients with a Fontan circulation need maintenance of a CVP that is sufficient to drive its passive transpulmonary blood flow and achieve hemodynamic stability [4]. However, in the present case, circulatory collapse was not observed, although low CVP values (<5 mmHg) continued for approximately 20 min. A CVP transducer was set at the same height as the patient’s heart and was fixed at that level throughout the operation. The intraoperative table rotations for the surgical procedure might slightly affect CVP values. However, the temporal changes of CVP values, decreasing during acute bleeding and recovering after fluid resuscitation, suggest that CVP values reflected the patient’s circulatory condition. We compared the ventricular systolic function and diastolic dimension examined by TTE before and after the operation. Similar adequate ventricular systolic performance and diastolic dimension were observed with the CVP value of 13 mmHg on postoperative day 3. Therefore, the intraoperative CVP value of 10–15 mmHg was considered an appropriate target for circulatory management of the patient. A previous study showed that a low hematocrit resulted in a decrease in pulmonary vascular resistance [8]. The decreased hematocrit that occurred in the present case probably supported the pulmonary circulation. Nevertheless, we cannot definitively explain why ABP was maintained at such a low CVP (<5 mmHg) in this case.

The SVV value is calculated by assessing the stroke volume changes due to the positive pressure ventilation cycle, and it increases sensitively in hypovolemic conditions even though ABP and HR do not change in cases with a normal circulation [7]. The cut-off value of SVV for predicting fluid responsiveness is thought to be 12–13% [7]. However, in our patient, only a slight rise in SVV from 5 to 8% was observed during acute bleeding. The effects of positive pressure ventilation on Fontan hemodynamics have not been evaluated systematically [2]. In a Fontan circulation, the mechanism of respiratory stroke volume change is completely different from that in the normal circulation because of the absence of interventricular interaction. Stroke volume changes due to respiration are derived from only the release of the large reservoir of blood stored in the pulmonary vasculature system [2]. In addition, the morphologic right ventricle in the present HLHS case may not show an obvious change of stroke volume on respiration even in hypovolemic conditions. Therefore, SVV may have underestimated the change of volume status in patients with a Fontan circulation.

Transesophageal echocardiography was not used because the patient’s heart function was preserved preoperatively. In addition, the patient was placed in the right semilateral decubitus position and she was facing away from the anesthesia apparatus. TTE could be useful for the assessment of cardiac function and fluid management even in this situation. We prepared TTE in case of circulatory collapse. However, ABP and HR of the present case were maintained within an acceptable range during surgery. Thus, we did not use TTE for intraoperative management.

## Conclusions

CVP decreased sharply, whereas SVV rose only slightly during acute bleeding in the present case. CVP monitoring may have been useful for the management of an acute bleeding case with a Fontan circulation but SVV may not have been reliable. More patients with a Fontan circulation will undergo noncardiac surgeries; therefore, appropriate circulatory monitoring in these patients should be further investigated.

## Abbreviations

ABP: Arterial blood pressure
AVM: Arteriovenous malformation
CI: Cardiac index
CVP: Central venous pressure
HLHS: Hypoplastic left heart syndrome
HR: Heart rate
SVV: Stroke volume variation
TTE: Transthoracic echocardiography
